# Declines in Student Obesity Prevalence Associated with a Prevention Initiative — King County, Washington, 2012

**Published:** 2014-02-21

**Authors:** Eli Kern, Nadine L. Chan, David W. Fleming, James W. Krieger

**Affiliations:** 1 Public Health – Seattle & King County

The United States has invested heavily, through public and private sector initiatives, in actions to prevent youth obesity by promoting healthy eating and physical activity. This report documents recent trends in youth obesity in King County, Washington, which implemented a Communities Putting Prevention to Work (CPPW) obesity prevention initiative during 2010–2012, including a school-based component. Similar large-scale obesity prevention initiatives did not occur elsewhere in Washington. Beginning in 2004, the Washington State Department of Health began monitoring youth obesity through the biennially administered Washington State Healthy Youth Survey (HYS). Based on data from this survey, neither King County nor the rest of Washington showed statistically significant changes in obesity prevalence in 2006, 2008, and 2010, relative to 2004. In 2012, however, King County youth obesity prevalence showed a statistically significant decrease, while no change occurred in the remainder of the state. Within King County, CPPW was implemented only in low-income school districts to address geographic inequities in obesity rates. Analysis within King County comparing CPPW and non-CPPW school districts before and after the intervention (2010 versus 2012) revealed a statistically significant decline in obesity prevalence in CPPW schools yet no change in non-CPPW schools. This decline in CPPW schools was significantly different than in non-CPPW schools. These findings suggest that school-based policy, systems, and environment changes might help reduce youth obesity, warranting further evaluation of short- and long-term impacts on population health.

The analysis used data from the HYS (1), a school-based survey analogous to the Youth Risk Behavior Survey, conducted each even-numbered year during 2004–2012. The Washington State Department of Health used self-reported height and weight data from the survey (asked only of respondents in grades 8, 10, and 12) to calculate body mass index (BMI), then used standard BMI-for-age charts to classify each respondent as obese or not obese. Obesity was defined as a BMI equal to or greater than the 95th percentile for children of same age and sex in year 2000 national growth charts (2). Survey response rates for grades 8, 10, and 12 combined from 2004 to 2012 ranged from 63% to 71% (approximately 34,000 respondents per survey year) and 61% to 67% (approximately 18,500 respondents per survey year) for King County and the rest of Washington, respectively. Data were weighted to be representative of school enrollment by year, grade, and sex.

The 2004–2012 obesity trend among students (grades 8, 10, 12 combined) in King County was compared with the trend in the rest of Washington. Within King County, the 2010 to 2012 change in obesity prevalence in school districts that received CPPW interventions was compared with non-CPPW districts. For comparison of King County with the rest of Washington, logistic regression analysis was used to assess the significance of obesity trends from 2004 to 2012, and the statistical interaction of geography and year was used to test for a significant difference in trends. For comparison of CPPW with non-CPPW school districts, the aim was to assess the impact of the intervention. Logistic regression analysis was used to estimate the 2010 to 2012 change in the odds of obesity for each group, and the statistical interaction of group and year was used to test for a significant difference in the change in the odds of obesity across groups. For the King County versus rest of Washington analysis, race/ethnicity, maternal education, and sex were evaluated as potential confounders by 1) assessing associations with year and obesity (chi-squared test; p<0.05 identifies potential confounder) and 2) for those associated with both, including these in logistic regression models to compare crude and adjusted trends (≥10% change in crude estimate identifies confounder). Two-tailed p-values <0.05 were considered to indicate significance in all statistical tests.

Among students in both King County and the rest of Washington State, no statistically significant changes were observed in the prevalence of obesity from the baseline 2004 HYS survey through 2010. In 2012, for the first time, obesity prevalence in King County showed a statistically significant decrease, from 9.5% in 2004 to 7.9% in 2012, with the odds of a student being obese in 2012 being 10% less than in 2004 (odds ratio [OR] = 0.90; 95% confidence interval [CI] = 0.82–0.98). In contrast, among students in the rest of Washington, obesity prevalence was stable from 2004 to 2012 (Figure 1, Table 1). The difference in the change over time in obesity prevalence between King County and the rest of Washington was significant (King County students saw greater reduction; p-value for interaction = 0.02). No evidence of confounding was identified; neither maternal education nor sex distributions changed over time in these populations, and although race/ethnicity distributions did change over time, obesity trends adjusted for race/ethnicity were similar (<10% change) to crude trends.

The CPPW initiative was implemented in 2010. CPPW students represented 57% of King County students, were more likely than non-CPPW students to be eligible for free and reduced price lunch (44% versus 17%), and had higher baseline obesity prevalence (Figure 2, Table 2). Among students in King County’s non-CPPW school districts, obesity prevalence was stable from 2010 to 2012 (OR = 0.95; CI = 0.87–1.04). Among students in CPPW school districts, prevalence decreased significantly from 2010 to 2012, from 10.6% to 8.8%, and the odds of a student being obese in 2012 were 9.3% less than in 2010 (OR = 0.91; CI = 0.84–0.98) (Figure 2). These changes were temporally associated with school-based CPPW interventions. Comparing CPPW and non-CPPW students, the 2010 to 2012 change in obesity was significantly different (CPPW districts saw greater reduction; p=0.045 for interaction term). Before the CPPW intervention in 2010, obesity prevalence was stable in the CPPW districts, whereas it declined in the non-CPPW districts.

## Editorial Note

This report demonstrates a temporal and spatial association between declines in self-reported youth obesity and implementation of a CPPW project during 2010–2012. King County CPPW focused its efforts on low-income school districts and communities because community health assessment data indicated that the prevalences of obesity, poor nutrition, and physical inactivity were disproportionately high relative to higher-income communities. Although data were available only for students in grades 8, 10, and 12, CPPW school district interventions reached all students (grades K through 12) and included implementation of nutrition standards for school meals, student-led healthy eating and active living promotional campaigns, farm-to-school initiatives, high-quality physical education, nutrition and culinary training for school cafeteria staff, and participation in community health coalitions (3). Youth obesity prevalence monitored from 2004 first showed a statistically significant decline in King County in 2012 after implementation of CPPW but not in the rest of Washington, where no comparable initiatives took place, and within King County in CPPW school districts but not in non-CPPW districts. In CPPW school districts, obesity prevalence dropped by 17%, and a student’s odds of being obese fell by 9.3% from 2010 to 2012 (OR = 0.91), whereas obesity prevalence in non-CPPW school districts remained stable during this period.

These decreases in youth obesity prevalence are consistent with trends reported from other metropolitan sites that also have implemented robust obesity prevention initiatives (4). This report extends these observations by demonstrating both a temporal association with CPPW implementation and a spatial association with the location of CPPW investment.

The findings in this report are subject to at least five limitations. First, weight and height were self-reported; thus, the findings are subject to recall and response bias. Additionally, self-reported weight and height tend to underestimate BMI (5); however, it is unlikely that the degree of underestimation would change during the study period, and thus, this would not affect analysis of temporal trends. Second, it was not possible to fully control for factors that could confound obesity trends, such as differential changes over time in household income. Third, the limited number of time points and sample size precluded a more robust use of time-series analytic methods and stratified or multivariate analyses to assess interactions and address confounding. Fourth, this is a preliminary finding describing a short-term trend. Finally, this is an observational study; the findings cannot be used to establish causality. If in fact CPPW did in part reduce youth obesity prevalence, other factors also might have contributed to the decreases in King County and CPPW school districts obesity rates, such as non-CPPW community-level healthy eating and active living programs and secular population-wide obesity trends.

CDC has prioritized obesity prevention as one of its 10 “winnable battles.”* CPPW was a targeted CDC intervention to prevent obesity by promoting healthy eating and physical activity. These findings suggest that focused and comprehensive policy, systems, and environment change interventions can reduce obesity in youth. Future analysis of HYS data, as they become available, will support assessment of CPPW’s longer-term impacts on youth obesity. Continued community-level interventions paired with robust epidemiologic, cost and process evaluations might prevent obesity, provide the opportunity to learn more about how these comprehensive interventions work, and identify which elements are most cost-effective in reducing obesity and improving population health across various settings (6,7).

What is already known on this topic?

Early signs of declines in youth obesity have been reported from localities and states that have implemented robust obesity prevention initiatives.

What is added by this report?

By 2012, for the first time, self-reported youth obesity prevalence in King County, Washington, saw a statistically significant decrease from its 2004 baseline prevalence, from 9.5% in 2004 to 7.9% in 2012, after a Communities Putting Prevention to Work project was implemented in the county’s low-income school districts from 2010 to 2012.

What are the implications for public health practice?

School-based policy, systems, and environment changes might be important elements of a comprehensive obesity prevention strategy.

## Figures and Tables

**FIGURE 1:**
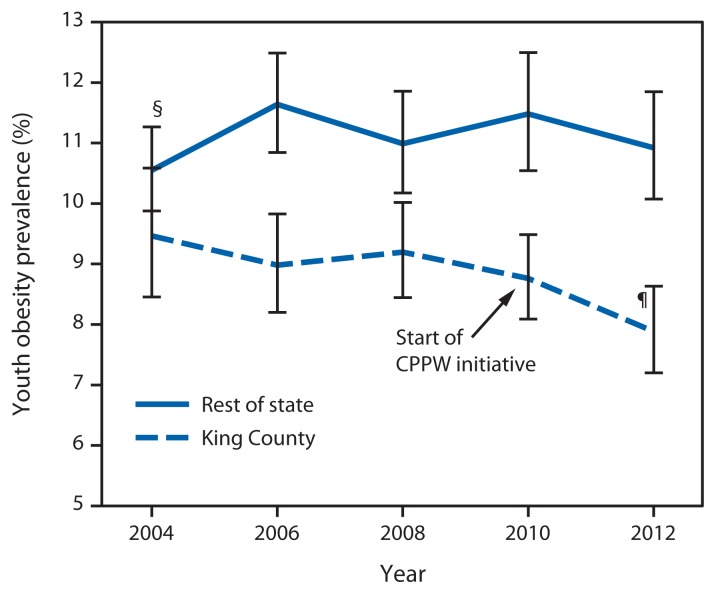
Prevalence of youth* obesity in King County, Washington, compared with the rest of the state, 2004–2012^†^ **Abbreviation:** CPPW = Communities Putting Prevention to Work. * Students in grades 8, 10, and 12 are combined. ^†^ Data are weighted to be representative of school enrollment by year, grade, and sex. ^§^ 95% confidence interval. ^¶^ Obesity trend for 2004–2012 shows statistically significant decline (p<0.05).

**FIGURE 2:**
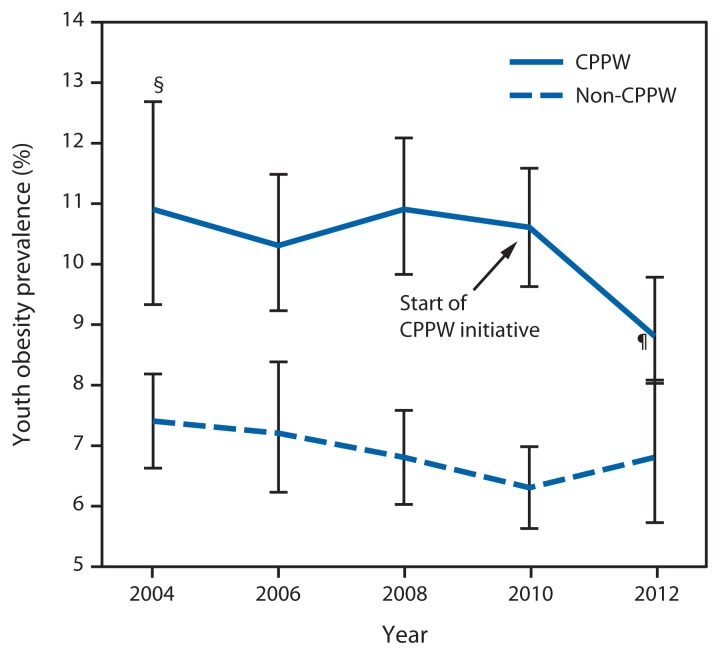
Prevalence of youth* obesity, by school district participation in the Communities Putting Prevention to Work (CPPW) initiative — King County, Washington, 2004–2012^†^ * Students in grades 8, 10, and 12 are combined. ^†^ Data are weighted to be representative of school enrollment by year, grade, and sex. ^§^ 95% confidence interval. ^¶^ Change in obesity since 2010 statistically significant (p<0.05).

**TABLE 1:** Prevalence of youth* obesity in King County compared with the rest of Washington state, 2004–2012†

	King County		Rest of state	
		
Year	Prevalence (%)	(95% CI)	Prevalence (%)	(95% CI)
2004	9.5	(8.4–10.6)	10.6	(9.9–11.3)
2006	9.0	(8.2–9.9)	11.6	(10.8–12.5)
2008	9.2	(8.4–10.0)	11.0	(10.2–11.9)
2010	8.8	(8.1–9.5)	11.5	(10.5–12.5)
2012	7.9§	(7.2–8.7)	10.9	(10.1–11.9)

**Abbreviation:** CI = confidence interval.

* Students in grades 8, 10, and 12 are combined.

† Data are weighted to be representative of school enrollment by year, grade, and sex.

§ Obesity trend for 2004–2012 shows statistically significant decline (p<0.05).

**TABLE 2:** Prevalence of youth* obesity, by school district participation in the Communities Putting Prevention to Work (CPPW) initiative — Washington state, 2004–2012†

	CPPW districts		Non-CPPW districts	
		
Year	Prevalence (%)	(95% CI)	Prevalence (%)	(95% CI)
2004	10.9	(9.3–12.7)	7.4	(6.6–8.2)
2006	10.3	(9.2–11.5)	7.2	(6.2–8.4)
2008	10.9	(9.8–12.1)	6.8	(6.0–7.6)
2010	10.6	(9.6–11.6)	6.3	(5.6–7.0)
2012	8.8§	(8.0–9.8)	6.8	(5.7–8.1)

**Abbreviation:** CI = confidence interval.

* Students in grades 8, 10, and 12 are combined.

† Data are weighted to be representative of school enrollment by year, grade, and sex.

§ Change in obesity since 2010 is statistically significant (p<0.05).

## Footnotes

* Information available at http://www.cdc.gov/winnablebattles/obesity/index.html.
